# Cardiac Repair With Echocardiography-Guided Multiple Percutaneous Left Ventricular Intramyocardial Injection of hiPSC-CMs After Myocardial Infarction

**DOI:** 10.3389/fcvm.2021.768873

**Published:** 2021-11-04

**Authors:** Xun Wu, Di Wang, Kele Qin, Chukwuemeka Daniel Iroegbu, Kun Xiang, Yuanjing Zhou, Qing Guan, Weijie Tang, Jun Peng, Jianjun Guo, Jinfu Yang, Chengming Fan

**Affiliations:** ^1^Department of Cardiovascular Surgery, Second Xiangya Hospital, Central South University, Changsha, China; ^2^Hunan Provincial Key Laboratory of Cardiovascular Research, Changsha, China; ^3^Hunan Fangsheng Pharmaceutical Co., Ltd., Changsha, China

**Keywords:** myocardial infarction, echocardiography-guided, intramyocardial injection, stem cells, therapy

## Abstract

**Objective:** We investigated the potency of cardiac repair based on echocardiography-guided multiple percutaneous left ventricular intramyocardial injection of human induced pluripotent stem cell-derived cardiomyocytes (hiPSC-CMs) after myocardial infarction (MI).

**Methods:** Mice with surgically induced MI were randomly divided into three groups (*n* = 8 in each group) and subjected to echocardiography-guided percutaneous left ventricular infarcted border injection of hiPSC-CMs (single dose; 10 μl 3 × 10^5^ cells) or repeated injections of hiPSC-CMs at post-MI weeks 1 and 2 (multiple doses). The sham group of animals underwent all surgical procedures necessary for MI induction except for ligation. Then 4 weeks after MI, heart function was measured with transthoracic echocardiography. Engraftment was evaluated through the detection of human-specific cardiac troponin T. Infarct size and collagen volume were calculated with Sirius Red/Fast Green staining. Angiogenesis was evaluated with isolectin B4 staining. Cardiac remodeling was evaluated from the cardiomyocyte minimal fiber diameter in the infarcted border zone. Apoptosis was detected *via* TdT-mediated dUTP Nick-End Labeling (TUNEL) staining in cardiomyocytes from the infarcted border zone.

**Results:** No mice died after echocardiography-guided percutaneous left ventricular intramyocardial injection. hiPSC-CMs were about nine-fold higher in the multiple-dose group at week 4 compared to the single-dose group. Multiple-dose transplantation was associated with significant improvement in left ventricular function, infarct size, angiogenesis, cardiac remodeling, and cardiomyocyte apoptosis.

**Conclusion:** Echocardiography-guided multiple percutaneous left ventricular intramyocardial injection is a feasible, satisfactory, repeatable, relatively less invasive, and effective method of delivering cell therapy. The delivery of hiPSC-CMs indicates a novel therapy for MI.

## Introduction

The leading cause of death from coronary heart disease worldwide is acute myocardial infarction (MI) (1). Myocardial cells in the infarcted area die quickly when ischemia and hypoxia occur. The injured heart tissue then becomes scar tissue, which has few mechanical or electrical properties and ultimately compromises cardiac function. Cardiac regeneration in the mammalian adult heart is very limited. After myocardial infarction, the viable myocardium is not enough to maintain sufficient cardiac output, which may eventually lead to heart failure (2). Treating heart failure is challenging because conventional treatments, including drug therapy and interventional therapy, are unable to restore heart function; in the late stages of heart failure, the only treatment option is heart transplantation or the use of ventricular assist devices (3, 4). However, organ shortages, severe postoperative complications, and the side effects of long-term immunosuppressive therapy limit the use of these therapies (5). Therefore, early treatment of MI is of great importance, and the use of human induced pluripotent stem cell-derived cardiomyocytes (hiPSC-CMs) is considered a promising method of repairing the damaged heart.

The major discovery of induced pluripotent stem cells makes it possible to directly reprogram adult cells into pluripotent cells (6), circumventing the ethical issues around the application of embryonic stem cells and rekindling hopes for repairing or regenerating damaged adult tissue, in particular in the heart. hiPSC-CMs are a promising source of cells for tissue regeneration after MI. However, the low survival rate after transplantation is a major obstacle to effective myocardial regeneration (7, 8). One of the main reasons for this is that the transplanted cells are in an environment characterized by ischemia and hypoxia and have limited nutrition (9). It is imperative to develop strategies to improve cell survival after transplantation to improve cardiac function after MI. Current approaches include the use of cell transplantation drugs, hypoxia and heat shock preconditioning (10–12), gene modification (13), treatment with prosurvival factors (14), and simultaneous microvascular and cell transplantation (15). Our previous studies have shown that hiPSC-CM transplantation can improve cardiac remodeling and promote angiogenesis in the infarct area (13, 16). In this study, we confirmed the feasibility of injecting multiple doses of percutaneous cells into left ventricular (LV) infarcted border intramyocardial tissue guided by echocardiography. We then explored whether multiple injections of cells could significantly increase the number of transplanted cells, reduce the area of cardiac scar tissue, and improve cardiac function compared to a single injection of cells, providing a new strategy for the clinical application of multiple injections of cells.

## Methods

### hiPSC-CM Culture and Cell Preparation

hiPSC-CMs, cardiomyocyte medium, cardiomyocyte plating medium, and cardiomyocyte digestive medium were provided by Help Stem Cell Innovations, Nanjing, China. hiPSC-CMs were cultured on petri dishes coated with cardiomyocyte plating medium and used after 60 days of maintaining and stabilizing after differentiation (17) (Supplementary Figure 1). Before use, hiPSC-CMs were digested with cardiomyocyte dissociation medium and resuspended in the culture medium, which had been preserved in a 37°C water bath. Cell transplantation was performed within a half hour of resuspension.

### Animal Model of MI and Cell Injection

**Figure 1:**
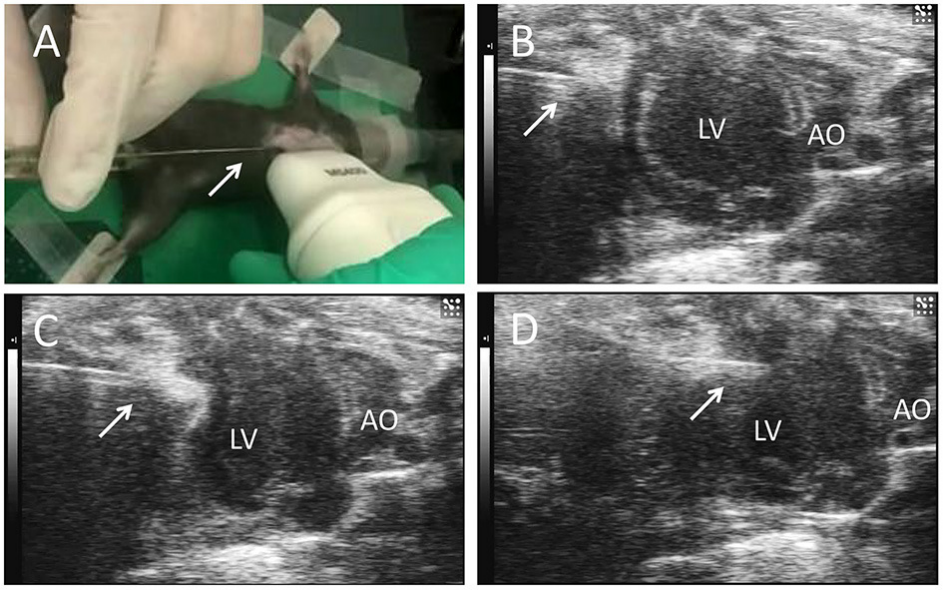
Echocardiography-guided percutaneous left ventricular intramyocardial injection. Mice were anesthetized and maintained *via* isoflurane (1.5−2%) inhalation. After depilation locally, and guided by transthoracic echocardiography, the needle (arrows) of a microsyringe was inserted below the xiphoid process **(A)** and along the upper margin of the liver **(B)**, entered the pericardial cavity **(C)**, and finally was injected into the anterior wall of the left ventricle **(D)** to deliver the cells. LV, left ventricle; AO, ascending aorta.

C57BL/6 mice induced MI by surgery. Feed 12-week-old mice at a temperature of 25°C and a humidity of 40–60% in a 12-h light/12-h dark cycle. Mice were anesthetized and maintained *via* isoflurane (1.5–2%) inhalation. After intubating and ventilating the mouse, perform thoracotomy in the third and fourth intercostal space on the left side of the mouse, and then ligate the left anterior descending coronary artery (LAD) with 8-0 non-absorbable sutures, and then close the chest muscles and skin. Then the surgical mice were randomly divided into three groups: single-dose cell therapy (SD) group and multiple-dose cell therapy (MD) group and MI-only group. The hiPSC-CM suspension (3 × 10^5^ cells in 10 μl) was percutaneously injected into 3 LV infarcted border intramyocardial sites (1 × 10^5^ cells per inject site) with a microsyringe (Cat. 84250; Hamilton Company) guided by a resolution micro-ultrasound system (Vevo 2100; Visualsonics) through the long-axis view (SD groups, Figure 1); the equivalent volume of PBS was injected in the same way (MI-only group). Except for coronary artery ligation, mice in the sham operation group received all the surgical operations required for MI treatment. After injection, mice were injected intraperitoneally with buprenorphine (0.1 mg/kg) every 12 h for 3 days, and carprofen (5 mg/kg) was injected intraperitoneally every 12 h for 12 days. To prevent graft rejection, we administered cyclosporine A (10 mg/kg/day) from 2 days before the injection of hiPSC-CMs until the mice were dissected. In addition to the first delivery, the MD group received repeated delivery of the same volume and concentration of hiPSC-CMs (3 × 10^5^ cells in 10 μl totally, and 1 × 10^5^ cells per inject site) at post-MI weeks 1 and 2 (MD groups, Figure 1). It is worth noting that the needle of the microsyringe was inserted below the xiphoid process of the sternum and entered the pericardial cavity along the upper margin of liver to avoid injury to the liver or lung (Supplementary Figure 2).

### Echocardiography

As mentioned earlier, pre-MI and post-MI mice were examined with echocardiography at 4 weeks. In short, mice were anesthetized with 1–2% isoflurane USP (Fluriso; VetOne) until the heart rate maintained between 500 and 600 bpm. A high-resolution micro-ultrasound system (Vevo 2100; Visualsonics) was used to acquire B-mode and 2D M-mode images from the long-axis and short-axis views of the mouse heart. Finally, the data obtained were analyzed. We calculated the LV ejection fraction (EF) and shortened fraction (FS) from the short-axis view using the improved Simpson's rule and Vevo analysis software. The ultrasound operator was unaware of the grouping of the experiment.

### Grafted Cell Number and Rate

The hiPSC-CMs were of human origin, engraftment was determined and calculated *via* histological assessments of cells that expressed the human variant of cardiac troponin T (hcTnT) and human nuclear antigen (HNA). Histological assessments of the engraftment rate were performed as described previously (13, 16). The quantification about the number of engrafted cells at week 4 after MI was done as follows (Supplementary Figure 3): Took the slice for staining in every 50th serial section of the whole heart. Then randomly captured 5 high magnified images per slice and counting the average grafted cell numbers per unit and set as A1/μm, and drawing then calculating the total engrated area of the slice and set as B1 μm^2^, thus the grafted number in this slice in A1 × B1. With the same method, we can get the grafted cell number per mouse heart = (A1 × B1+A2 × B2+.+A*n* × B*n*) × 50. The engraftment rate was calculated by dividing the total number of engrafted hiPSC-CMs by the number of delivery (3 × 10^5^ for SD group and 3 × 3 × 10^5^ for MD group) and expressed as a percentage.

### Infarct Size

4 weeks after MI, the mice were sacrificed by cervical dislocation and the heart was quickly excised and fixed with 4% paraformaldehyde at 4°C for 12 h, and then soaked in 30% sucrose at 4°C for 12 h. The processed heart is cryopreserved in optical cutting temperature compound. Coronal sections were cut from the apex of the heart to the base at 10 μm intervals. Then every 30 sections from the ligated site level to the apex were selected, fixed with Bouin solution, and stained with 0.04% Sirius Red to identify cardiac collagen tissue and 0.1% Fast Green to identify cardiac non-collagen tissue. The digital photos of the slices were analyzed using ImageJ software, and the myocardial infarction area was calculated according to the following formula: Infarct area% = [(scar circumference × thickness of each minor axis)/(short axis left ventricle length × short axis thickness)] × 100%. Eight mice were evaluated in each group.

### Detection of Apoptotic Cells by TUNEL Assay

The apoptotic cells were fluorescently stained with the *in situ* cell death detection kit (Roche), and the apoptosis of the cells was detected and quantified by a fluorescent microscope. Heart samples from the mice were collected 4 weeks after MI. Frozen sections were washed 3 times in DPBS pH 7.4, and then fixed with 4% PFA for 20 mins at room temperature. The labeling reaction of the TUNEL reaction mixture was carried out in accordance with the manufacturer's instructions. Finally, the nuclei were stained with 4,6-diamidino-2-phenyl-indole (DAPI; vector) and mounted.

### Immunostaining and Fluorescence Microscopy

The collected frozen sections of the heart were permeabilized with 0.5% Triton X-100 for 8 mins at room temperature, and blocked in DPBS and 10% donkey serum at pH 7.4 for 30 mins at 25°C. Sections, primary antibody, and blocking buffer (1.5% BSA, 100 mM glycine in PBS) were used overnight at 4°C at a dilution of 1:10 to 1:1,000. The primary antibodies used in the study are shown in Table 1. The secondary antibody was diluted 1:100 with the same blocking buffer, and incubated for 2 h at 25°C in the dark. DAPI (vector) is used for nuclear staining. The staining of the negative control includes only the secondary antibody. The sections were stained and analyzed with a fluorescence microscope (Olympus IX83).

**Table 1:** The primary antibodies used in the study.

**Primary antibody**	**Dilution**	**Source**	**Cat. no**.
**Antibodies and reagents**			
Human cardiac troponin T	1:100	Abcam	ab91605
Sarcomeric alpha actinin	1:100	Abcam	ab9465
Cardiac troponin T	1:100	Abcam	ab8295
Isolectin B4	1:100	Vector	FL-1201
Wheat germ agglutinin	1:1,000	Thermo Fisher	W11261

### Statistical Analysis

The data in this study are all expressed as mean ± standard error. All statistical calculations were performed using SPSS 21.0 (SPSS Institute). Perform an independent sample t test to determine the difference between the two groups. One-way ANOVA was performed with Dunn's multiple comparison tests to compensate for variables between multiple groups. *p* < 0.05 was considered statistically significant.

## Results

A total of 26 mice underwent surgically induced MI with ligation of the LAD. Another eight mice underwent all procedures except LAD ligation. Of the mice that underwent MI surgery, two died from perioperative complications. Thus, 24 MI mice received echocardiography-guided percutaneous LV intramyocardial injection of cells or PBS (Figure 1). No mice died after injection.

### Multiple Injections Increase the Potency of hiPSC-CMs for Cardiac Repair in MI Mice

**Figure 2:**
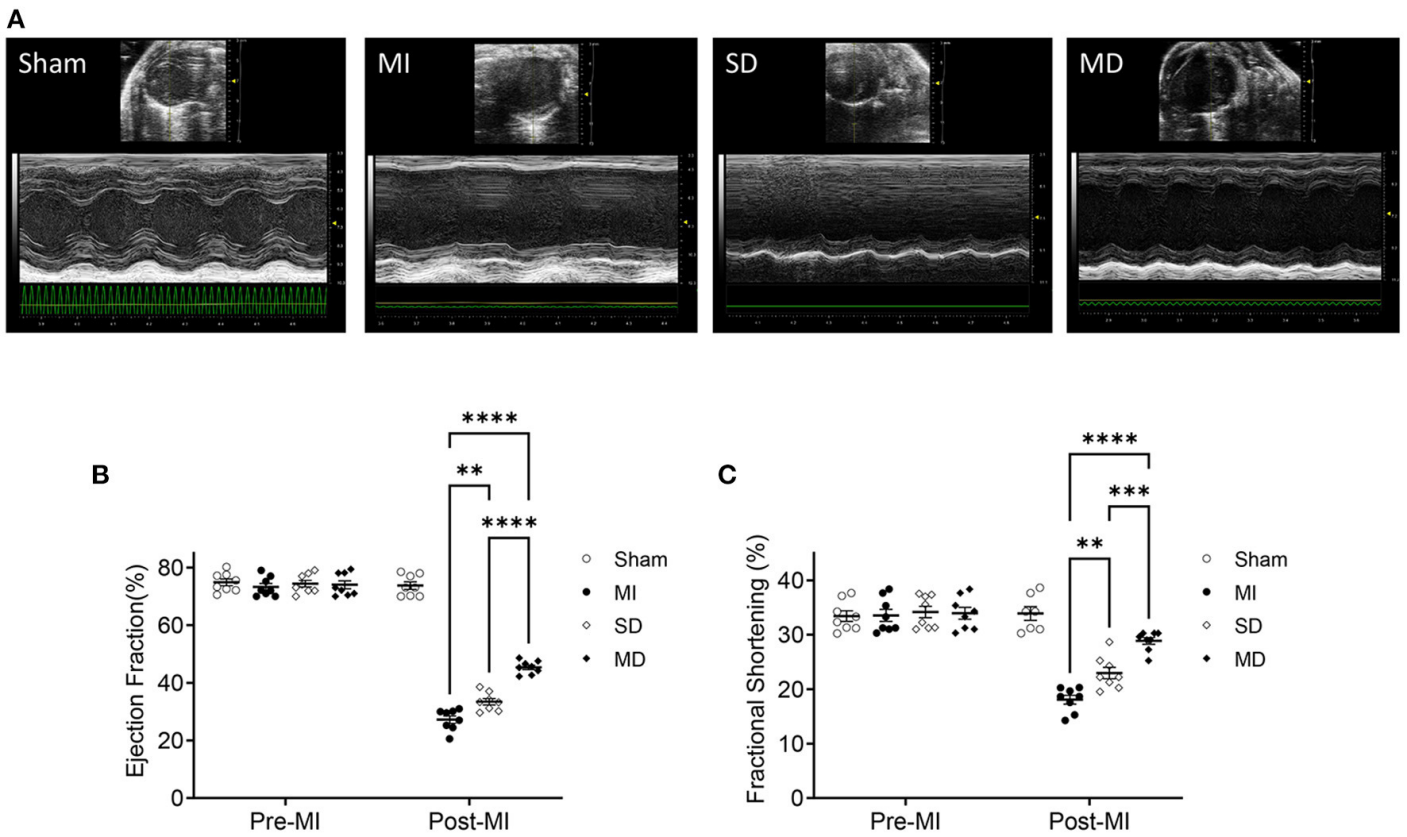
Assessment of cardiac function. Use high-resolution echocardiography to assess left ventricular function before MI induction (pre-MI) and 4 weeks after treatment **(A)**. The ejection fraction **(B)** and fractional shortening **(C)** are presented as absolute values. Data are means ± SE. Eight animals per group. One-way ANOVA with Dunn's multiple comparisons test. **p < 0.01; ***p < 0.001; ****p < 0.0001.

To determine whether multiple percutaneous intramyocardial injections of hiPSC-CMs can improve recovery from ischemic injury 4 weeks after transplantation, we used transthoracic echocardiography to evaluate LV function. Echocardiography (Figure 2A) showed that the LV EF of MI mice treated with PBS was significantly reduced by 40% (from 70 to 30%; Figure 2B) and that the ventricular FS was reduced by 14% (from 32 to 18%; Figure 2C). In the SD group, this remodeling process was attenuated, with increases of 5% in the EF and 4% in the FS (Figure 2C). It is worth noting that compared with the SD group, the EF and FS of the MD group increased significantly (Figures 2B,C).

### Multiple Injections Increase the Graft Size of hiPSC-CMs in MI Mice

**Figure 3:**
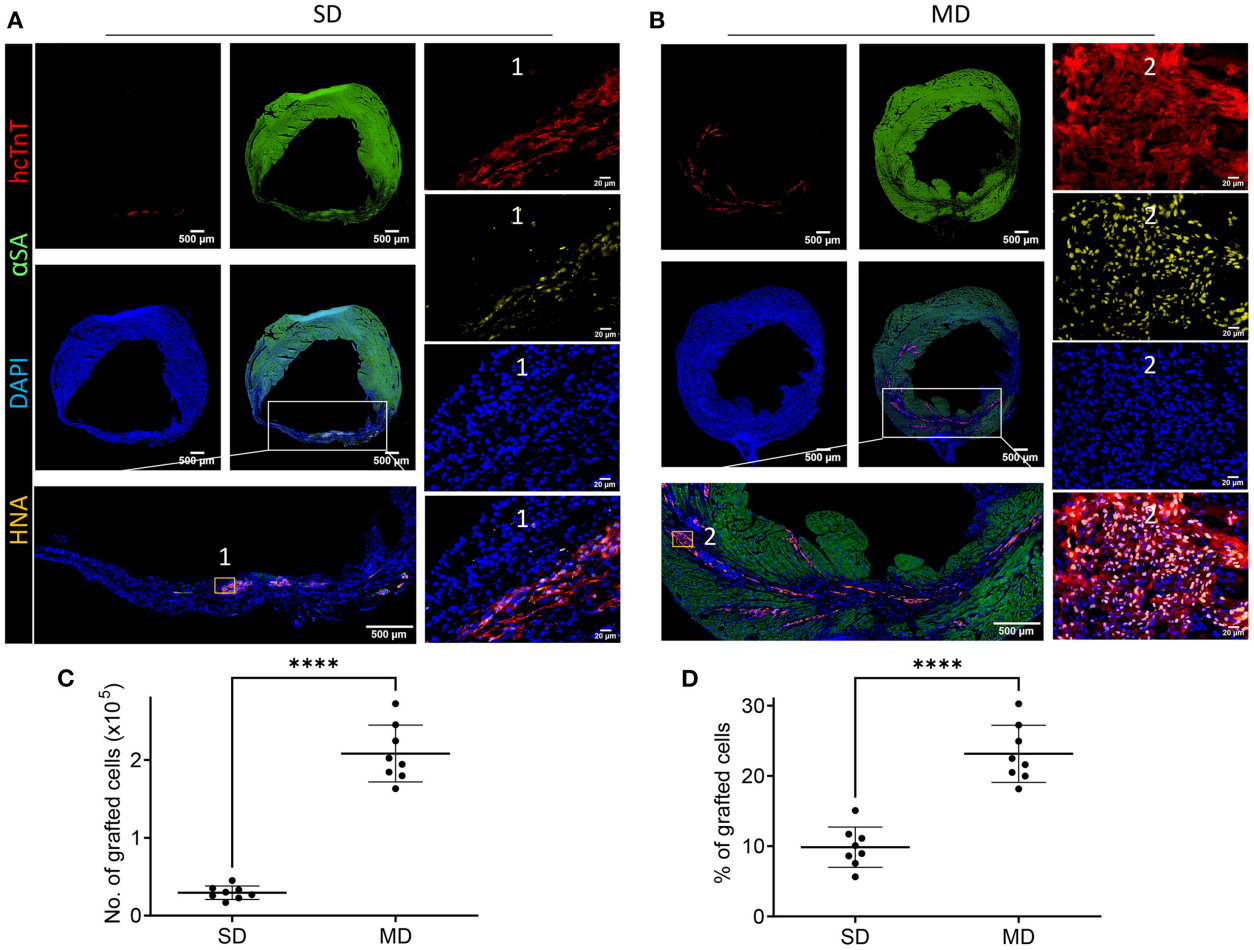
Detection of hiPSC-CMs. Four weeks after MI induction, serial sections of the hearts of mice injected with hiPSC-CM were stained for human cardiac troponin T (hcTnT) and human nuclear antigen (HNA), α-sarcomeric actin (αSA) and DAPI **(A,B)**. Transplanted cells were defined as those that expressed hcTnT and HNA and were quantified as the number **(C)** and percentage **(D)** of grafted cells. Each heart evaluates 20 parts, and each part randomly selects 5 visual fields for evaluation. Scale bar = 500 or 50 m as shown in the panels. Independent-samples t test. ****p < 0.0001.

One of the main challenges of heart cell therapy is its relatively low survival rate after transplantation. Therefore, we explored whether multiple percutaneous intramyocardial injections of hiPSC-CM can increase the number of transplanted cells and whether cardiac function can be significantly improved. Transplanted cells derived from human hiPSC-CM can be detected with human specific cardiac troponin T (hcTnT) and human nuclear antigen (HNA). The number of transplanted hiPSC-CMs was evaluated by immunofluorescence staining of heart tissue sections. Four weeks after MI, hiPSC-CMs were detected in the hearts of SD and MD animals (Figures 3A,B). We found that the number of transplanted cells in the MD group was significantly more than that in the SD group. The graft size was 10 times greater in the MD group than the SD group (Figure 3C). In addition, the percentage of grafted cardiomyocytes (the number of grafted cells divided by the total number of delivered cells) was also significantly higher in the MD group than in the SD group (Figure 3D). It is interesting that the hiPSC-CMs detected in the SD group were mainly distributed in the infarcted area (Figure 3A), whereas those in the MD group were widely distributed in the area of MI and the area of peri-infarction (Figure 3B).

### Multiple Cell Injections Decrease the Infarct Size

**Figure 4:**
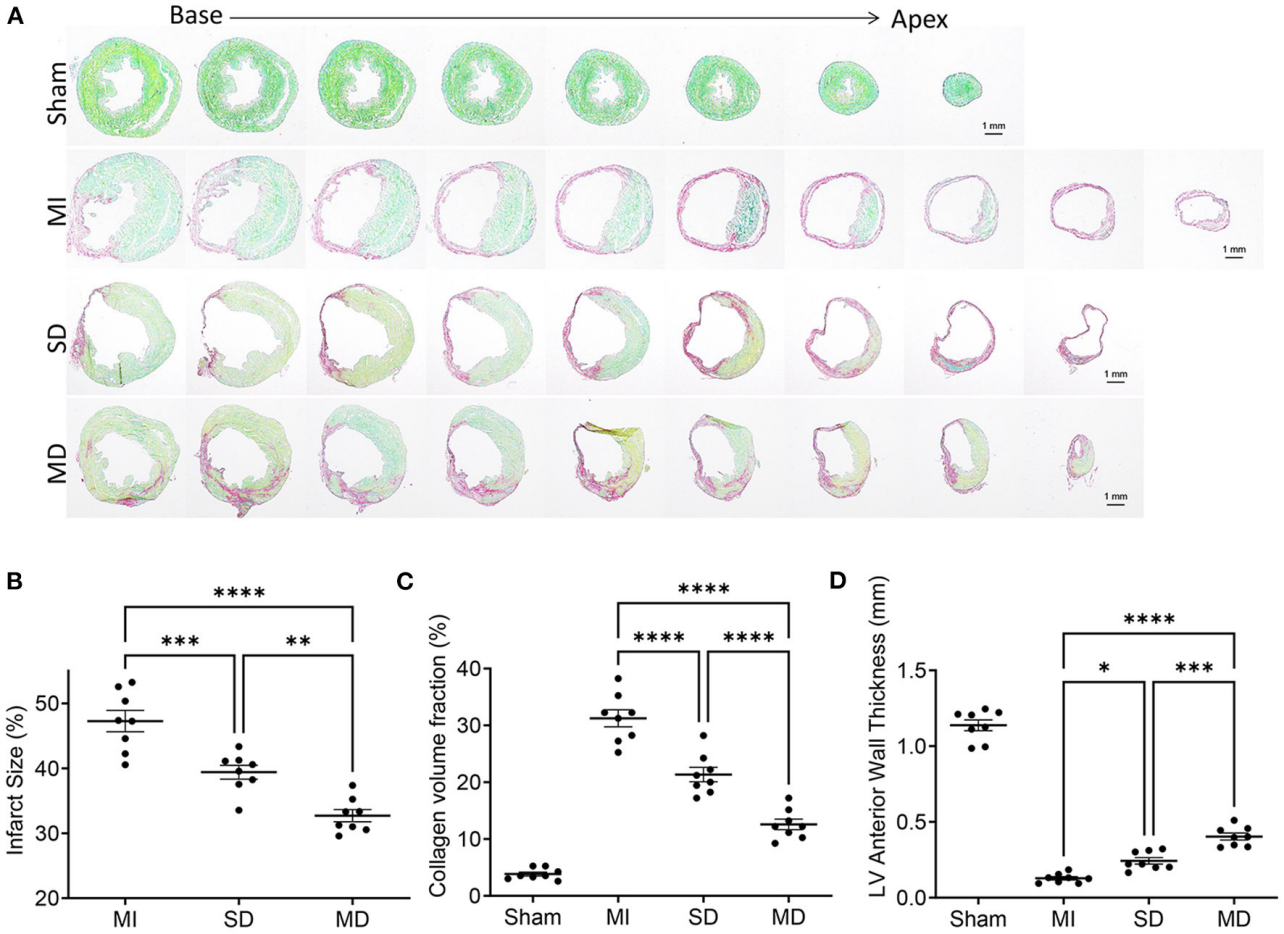
Evaluation of infarct size, collagen volume, and left ventricular morphology. The fibrotic (red; inactive) and non-fibrotic (green; active) areas of ventricular tissue sections were shown by Sirius Red/Fast Green histochemical staining **(A)** and quantification of infarct size **(B)**, collagen volume fraction **(C)**, and LV anterior wall thickness **(D)** in posttreatment week 4. Data are means ± SE. Eight animals per group. Scale bar = 1 mm. One-way ANOVA with Dunn's multiple comparisons test. *p < 0.05; **p < 0.01; ***p < 0.001; ****p < 0.0001.

Immunohistochemical staining of heart tissue from each group was performed to evaluate differences in infarct size, LV anterior wall thickness, and collagen volume fraction (Figure 4A). Histological evaluation showed that the infarct area (Figure 4B) and collagen volume fraction (Figure 4C) of the SD and MD groups were significantly smaller than those of the MI group only, and were most prominent in the MD group. The LV free wall thickness (Figure 4D) was significantly smaller in all three groups that underwent ligation than in the sham group and was significantly greater in the MD group than the SD group or MI-only group.

### Multiple Cell Injections Promote Neo-Angiogenic and Anti-apoptosis Responses After Transplantation

**Figure 5:**
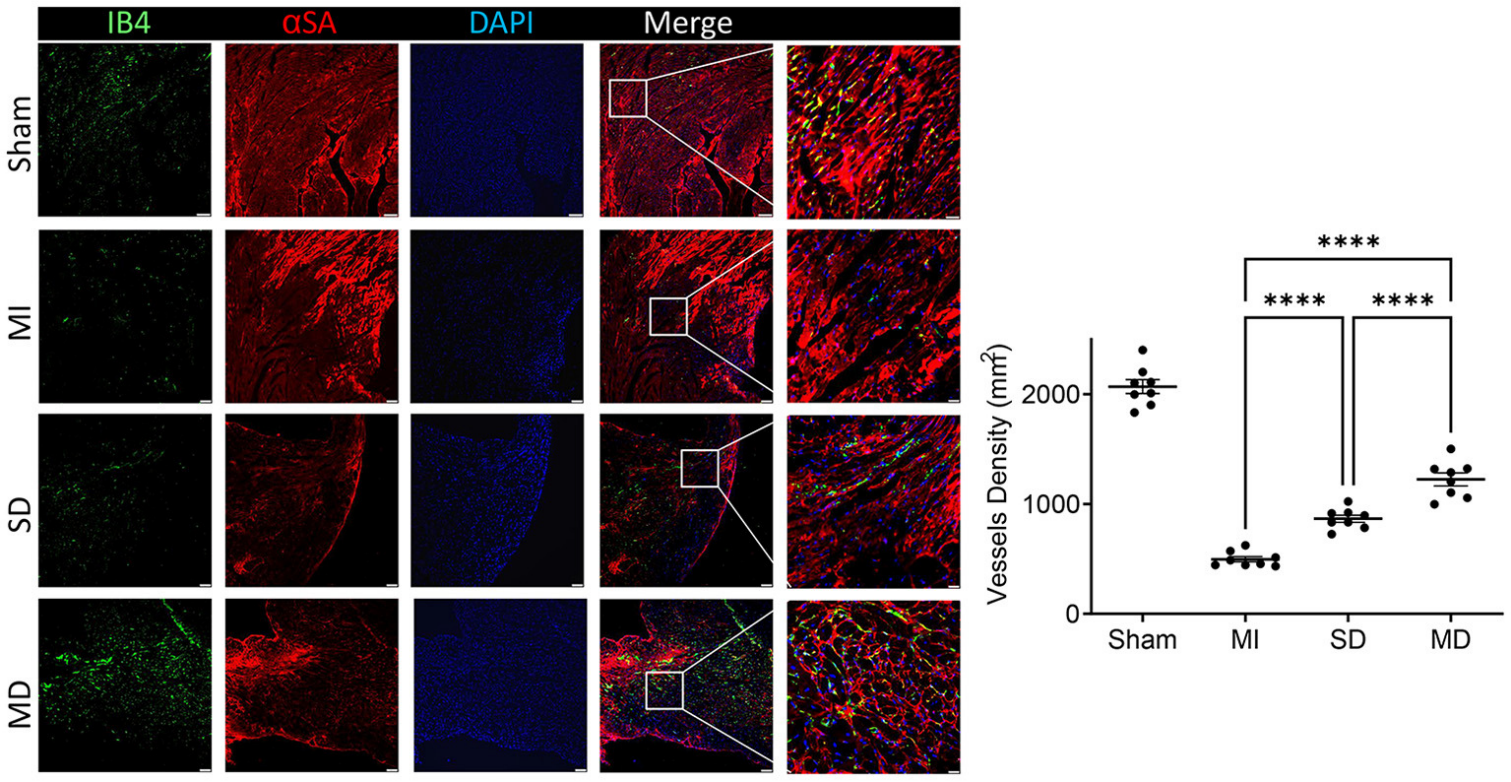
Assessment of angiogenesis. Histochemical staining was performed on serial frozen sections of sham-operated, MI, SD, and MD-treated mouse hearts that were sacrificed 4 weeks after MI induction to confirm the presence of IB4. The number of capillaries is quantified as the number of IB4-positive vascular structures in the area around the infarct per square millimeter. Data are means ± SE. Eight animals per group. Scale bar = 20 and 10 μm for the unenlarged and enlarged panels, respectively. One-way ANOVA with Dunn's multiple comparisons test. ****p < 0.0001.

**Figure 6:**
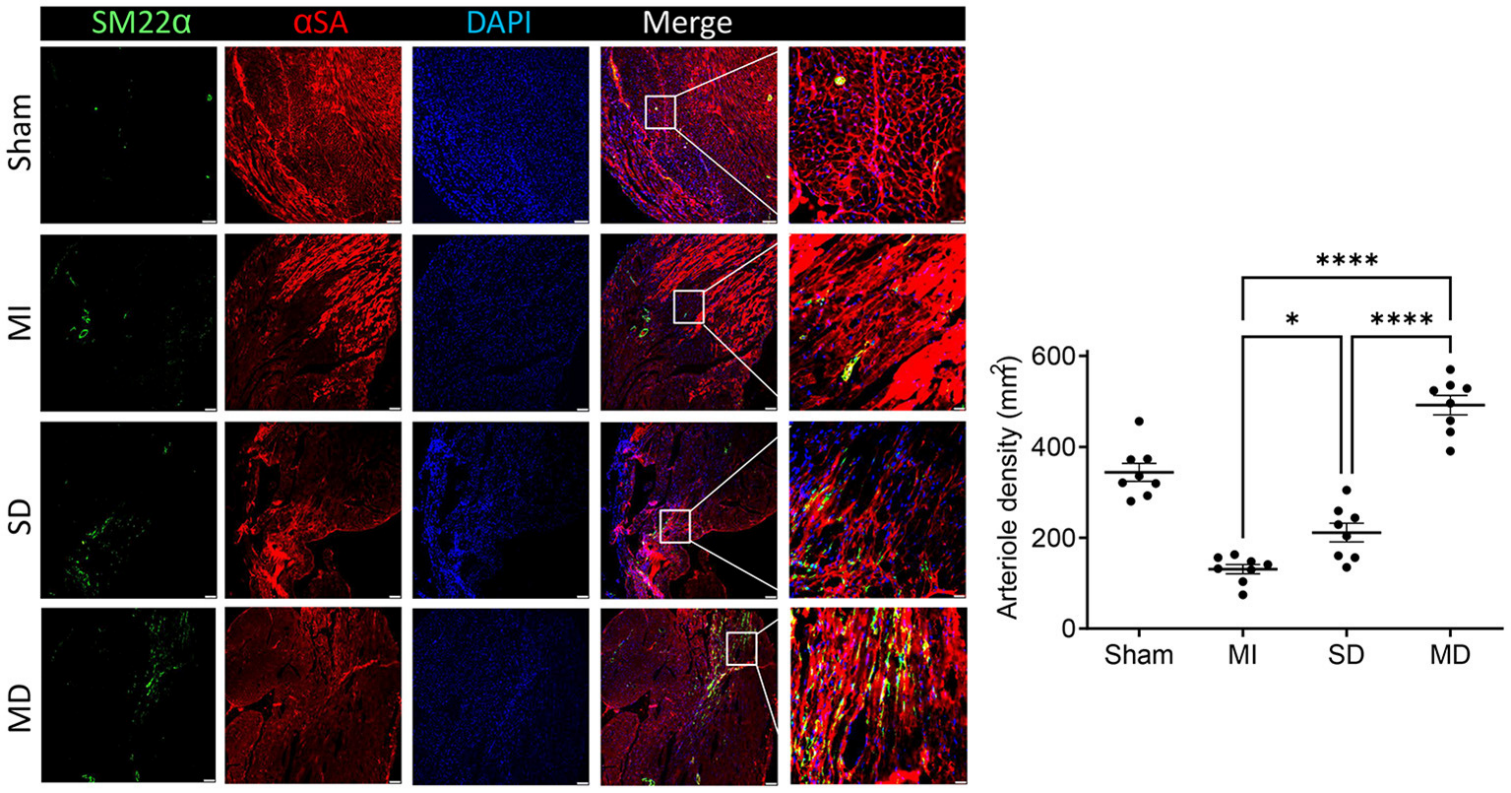
Assessment of arteriole density. Histochemical staining was performed on serial frozen sections of sham-operated, MI, SD, and MD-treated mouse hearts that were sacrificed 4 weeks after MI induction to confirm the presence of arterial structures. The number of artery is quantified as the number of SM22α-positive arterial structure in the area around the infarct per square millimeter. Data are means ± SE. Eight animals per group. Scale bar = 20 and 10 μm for the unenlarged and enlarged panels, respectively. One-way ANOVA with Dunn's multiple comparisons test. *p < 0.05; ****p < 0.0001.

**Figure 7:**
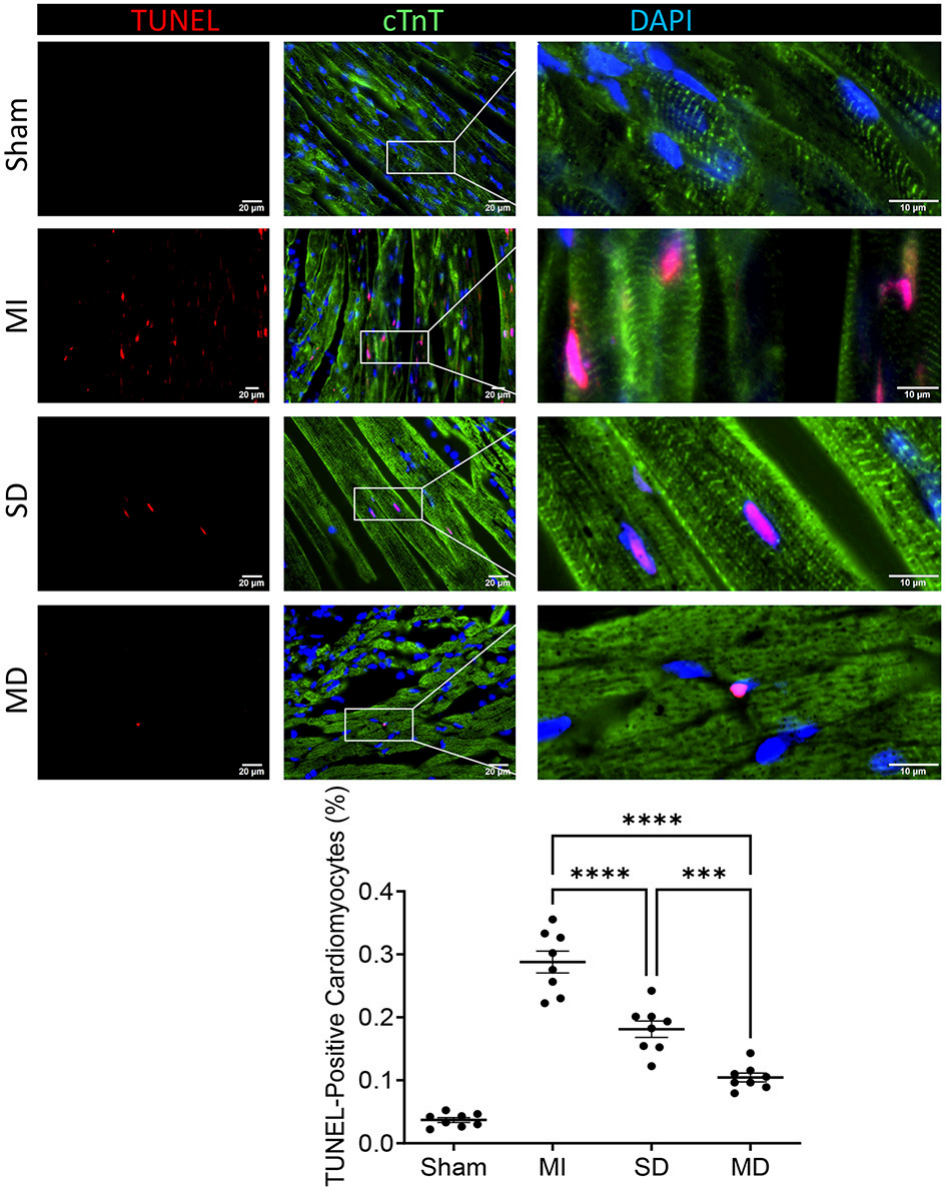
Assessment of cell apoptosis. After 4 weeks of different treatments, sections from the MI margin were stained with TUNEL to reveal apoptotic cardiomyocytes (red) and normal cardiomyocytes expressing cardiac troponin T (cTnT; green). The proportion of TUNEL-positive cells in total cells was calculated and expressed as a percentage. Data are means ± SE. Eight animals per group. Scale bar = 20 and 10 μm for the unenlarged and enlarged panels, respectively. One-way ANOVA with Dunn's multiple comparisons test. ***p < 0.001; ****p < 0.0001.

Angiogenesis and arterialization were evaluated with immunofluorescence staining of the endodermal cell marker isolectin B4 (IB4) and vascular smooth muscle cell marker SM22α in the peri-infarcted area. At 4 weeks postoperatively, the number of IB4 and SM22α expressing cells were significantly increased in the MD group than in the SD group or MI-only group (Figures 5, 6). We further evaluated cardiomyocyte apoptosis with TUNEL immunostaining (which labels the nuclei of all apoptotic cells). The prevalence of TUNEL-positive cardiomyocyte nuclei (Figure 7) was significantly lower in the hiPSC-CM-treated groups than in the MI-only group and was most prominent in the MD group.

### Multiple Cell Injections Alleviate LV Hypertrophy

**Figure 8:**
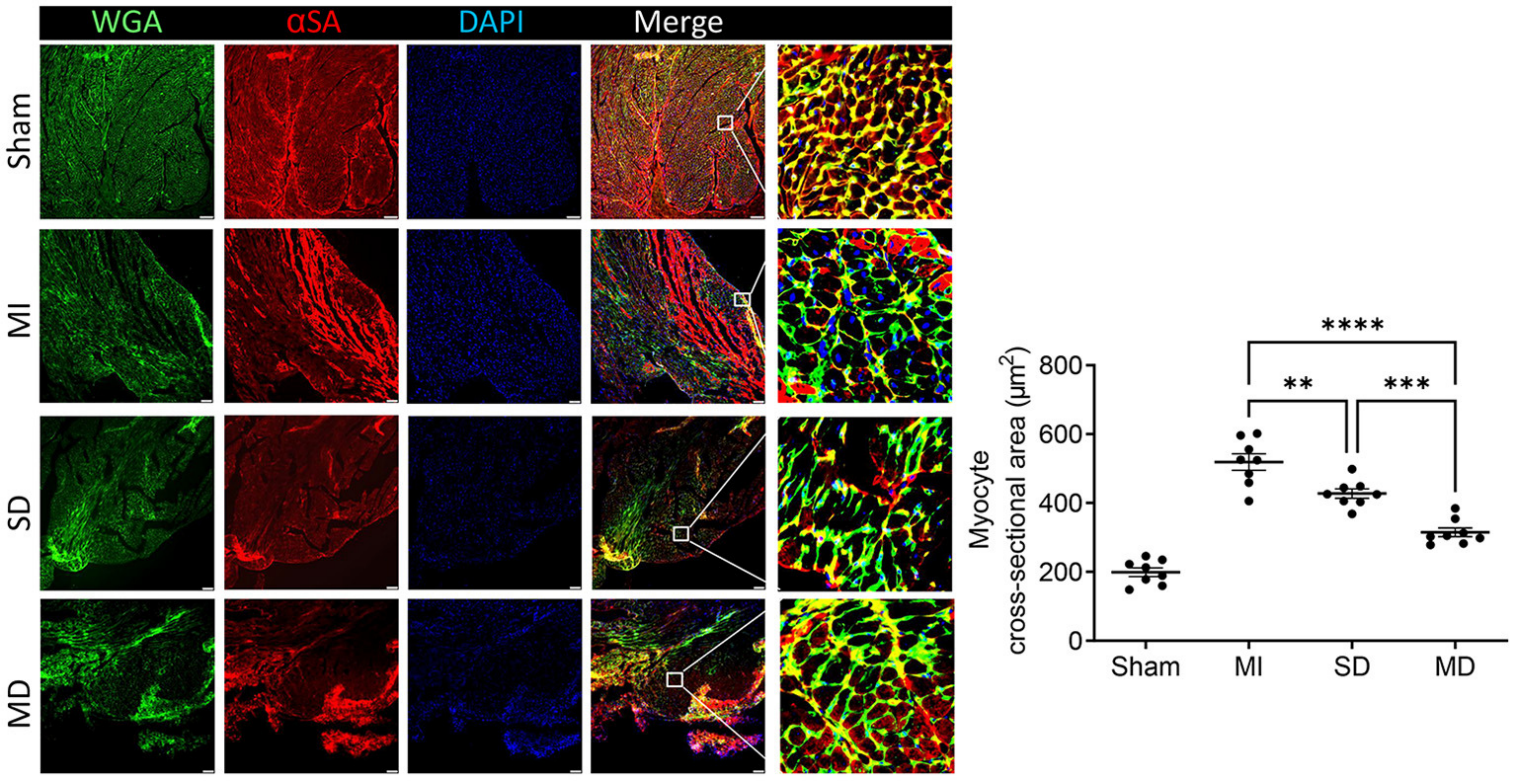
Assessment of left ventricular hypertrophy. Mice in each group were sacrificed 4 weeks after surgery, cardiomyocytes in the peri-infarcted area of the left ventricle were stained with wheat germ agglutinin and αSA, and nuclei were labeled by DAPI. The cross-sectional area of the cardiomyocytes was measured and is shown as an absolute value. Scale bar = 10 μm. One-way ANOVA with Dunn's multiple comparisons test. **p < 0.01; ***p < 0.001; ****p < 0.0001.

LV hypertrophy in each group was evaluated with wheat germ agglutinin and sarcomeric alpha actinin immunofluorescence staining of cardiomyocytes in the peri-infarcted area. The minimum fiber diameter was measured *via* the myocyte cross-sectional area (Figure 8) and was significantly more than in animals from all three groups that underwent ligation than in sham animals group. However, it was significantly smaller in the hiPSC-CM-treated groups than in the MI-only group and was most excellent in the MD group (Figure 8).

## Discussion

Stem cell-based cardiac repair is one of the most promising therapeutic approaches to regenerating damaged heart tissue (18). However, one of the main obstacles to the therapeutic efficacy of transplanted hiPSC-CMs is the small number of transplanted cells that remain and survive in the injection area (19). Existing research has tried several strategies to overcome the problem of low survival rate of transplanted cells, but these methods are not outstanding in improving cell survival rate (20–22). Recently, a variety of methods have been reported to enhance the survival of transplanted hiPSC-CMs to reduce myocardial infarction, including the addition of biologically active substances, genetic modification of cells, and the application of cell tissue patch (13, 16, 23–25). The problem of low survival rate of transplanted cells can be partially overcome by giving repeated doses of cells (20). If measured after a single dose, the benefits of cell therapy may be underestimated or even ignored; repeated doses are required to correctly evaluate the effectiveness of cell transplantation (22, 26). In rodents, repeated injections of cells are technically difficult and are associated with prohibitive mortality. In patients, the use of repeated intramyocardial or intracoronary cell injections is hampered by a variety of issues, including safety, financial, and regulatory issues. It is difficult enough for a single treatment to obtain regulatory approval; the barriers to multiple treatments are even greater (27). Echo-guided repeated intraventricular injection of cardiac progenitor cells is significantly more effective than a single injection (26). However, we believe that cell retention will benefit from intramyocardial delivery instead of intraventricular delivery. Percutaneous intramyocardial delivery is a safe and efficient method for local drug delivery (28). With the assistance of a mobile ultrasound machine, cell therapy can be performed at any time point. In the present study, we demonstrate for the first time that echocardiography-guided multiple percutaneous LV intramyocardial injection of cardiomyocytes significantly enhances the reparative properties of hiPSC-CMs. We found that multiple injections of hiPSC-CMs markedly improved the survival of the implanted cardiomyocytes (Figure 3), accompanied by increased contractile mass and a large replacement of scar tissue (Figure 4).

A number of studies have shown that compared with non-cardiac derivatives, cardiomyocytes derived from pluripotent stem cells can be implanted more effectively and improve heart function (14, 29, 30). Our previous study showed that transplanted hiPSC-CMs are electrically coupled with host cardiomyocytes (13). Therefore, the more cardiomyocytes that survive transplantation, the greater their contribution to cardiac output. In the present study, far more cardiomyocytes survived in the MD group than in the SD group. In terms of cardiac function, EF and FS values were both higher in the MD group than in the SD group. The number of transplanted cells in the MD group was 10 times that in the SD group, but the percentage of transplanted cells among total cells was only more than 2 times that of the SD group, which indicates that more transplanted cells more effectively resisted the apoptosis of the surrounding host cells. TUNEL staining in the marginal zone of MI confirmed that the TUNEL positive cells in the MD group were less than those in the SD group (Figure 7).

Many experiments have shown that the level of VEGF is positively correlated with an increase in microvessel density in the infarct area, which suggests that VEGF plays a role in myocardial remodeling and angiogenesis (31, 32). Although ischemia results in endogenous myocardial angiogenesis, it does not have the effect of maintaining normal capillary density (33). In our study, the MI-only group rarely had IB4-positive cells in the MI area; the MI-only group had the fewest IB4-positive cells, followed by the SD group and then the MD group, which suggests that the more hiPSC-CMs that are transplanted, the stronger the ability to form new blood vessels. Cardiomyocyte hypertrophy is an adaptive response of the heart to pressure overload, and it is also a common pathological feature during MI. In addition, the increased risk of heart failure and sudden cardiac death is closely related to cardiomyocyte hypertrophy (34). Our experiment confirms that transplanted hiPSC-CMs can improve cardiac remodeling and reduce cardiac hypertrophy and that the effect is far better in the MD group than in the other two groups.

## Study Limitations

The duration of our *in vivo* experiments was limited to 4 weeks, so the long-term fate and potential mechanisms of hiPSC-CMs transplanted over multiple doses remain unknown. Future investigations with larger animal models are needed to fully prove the mechanisms by which the administration of multiple doses of hiPSC-CMs improves myocardial recovery from MI and the long-term safety and effectiveness of this treatment.

## Conclusion

This is the first demonstration that repeated intramyocardial administrations of hiPSC-CMs are significantly more effective than a single dose. Echocardiography-guided multiple percutaneous LV intramyocardial injection of hiPSC-CMs is a viable, repeatable, relatively non-invasive, and effective method of delivering cell therapy. Our study also demonstrates that multiple intramuscular injections of hiPSC-CMs improve myocardial function, reduce the MI area, and reverse ventricular remodeling more than a single injection, which highlights the therapeutic potential of multiple injections of hiPSC-CMs for myocardial regeneration.

## Data Availability Statement

The original contributions presented in the study are included in the article/Supplementary Material, further inquiries can be directed to the corresponding authors.

## Ethics Statement

The animal study was reviewed and approved by Ethics Committee of the Second Xiangya Hospital of Central South University.

## Author Contributions

XW, DW, KQ, CI, KX, YZ, and QG carried out the data collection and assembly. XW and CF draft the manuscript. JP, JY, and CF carried out data analysis and interpretation. XW, JG, and CF carried out manuscript revising. CF carried out the conception and design. All authors read and approved the final manuscript.

## Funding

This work was supported by the Major Research Plan of the National Natural Science Foundation of China (No. 91539111 to JY); Key Project of Science and Technology of Hunan Province (No. 2020SK53420 to JY); Hunan Province Outstanding Postdoctoral Innovative Talent Project (2021RC2106 to CF).

## Conflict of Interest

JG and CF was employed by the company Hunan Fangsheng Pharmaceutical Co. Ltd. The remaining authors declare that the research was conducted in the absence of any commercial or financial relationships that could be construed as a potential conflict of interest.

## Publisher's Note

All claims expressed in this article are solely those of the authors and do not necessarily represent those of their affiliated organizations, or those of the publisher, the editors and the reviewers. Any product that may be evaluated in this article, or claim that may be made by its manufacturer, is not guaranteed or endorsed by the publisher.

## Supplementary Material

Supplementary Figure 1 The morphology of hiPSC-CMs on post cultured day 10 and 60. The cell size and sarcomere length were measured on post cultured day 10 and 60. Scale bar = 10 μm. ^***^*p* < 0.001. LV, left ventricle; AO, ascending aorta.

Supplementary Figure 2 The injection site during respiration. The heart was exposed during respiration, and part of the left ventricular tissue was uncovered by the lungs at both end-expiratory phase **(A)** and end-inspiratory phase **(B)**. The injection site is indicated by the arrow.

Supplementary Figure 3 Schematic diagram for the grafted cell number counting.

## Abbreviations

DAPI, 4: 6-diamidino-2-phenyl-indole
EF: ejection fraction
FS: fractional shortening
hcTnT: human variant of cardiac troponin T
hiPSC-CMs: human induced pluripotent stem cell-derived cardiomyocytes
IB4: isolectin B4
LAD: left anterior descending branch of the coronary artery
LV: left ventricle
MD: multiple dose
MI: myocardial infarction
SD: single dose
TUNEL: TdT-mediated dUTP Nick-End Labeling
VEGF: vascular endothelial growth factor.
