# Supplementary figures and images for: Cardiac Repair With Echocardiography-Guided Multiple Percutaneous Left Ventricular Intramyocardial Injection of hiPSC-CMs After Myocardial Infarction
Source: Front Cardiovasc Med. 2021 Nov 4;8:768873. doi: 10.3389/fcvm.2021.768873 (PMC8600116; doi:10.3389/fcvm.2021.768873)

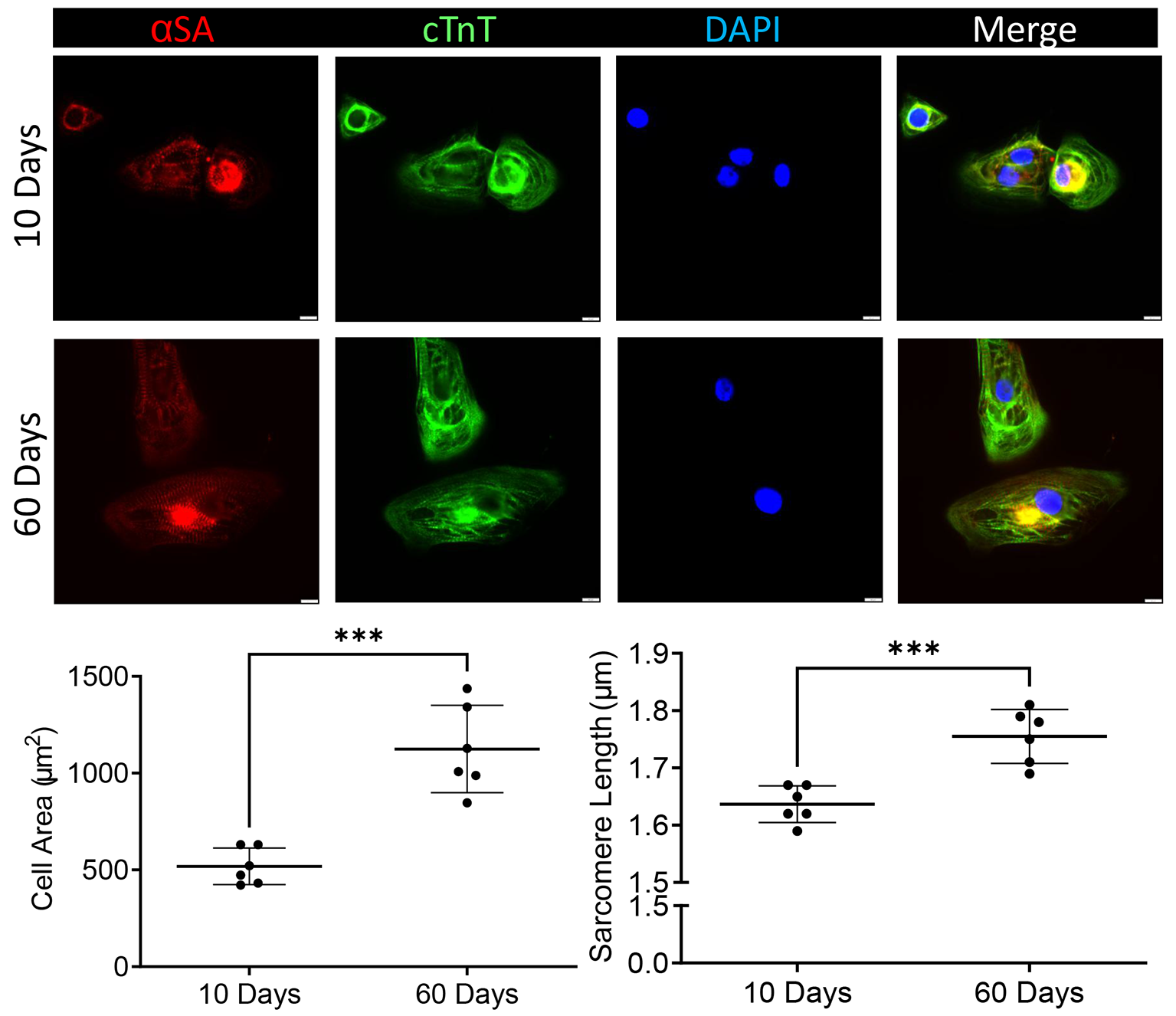

Supplement: Supplementary Figure 1 — The morphology of hiPSC-CMs on post cultured day 10 and 60. The cell size and sarcomere length were measured on post cultured day 10 and 60. Scale bar = 10 μm. ***p < 0.001. LV, left ventricle; AO, ascending aorta. [file Image_1.TIF]

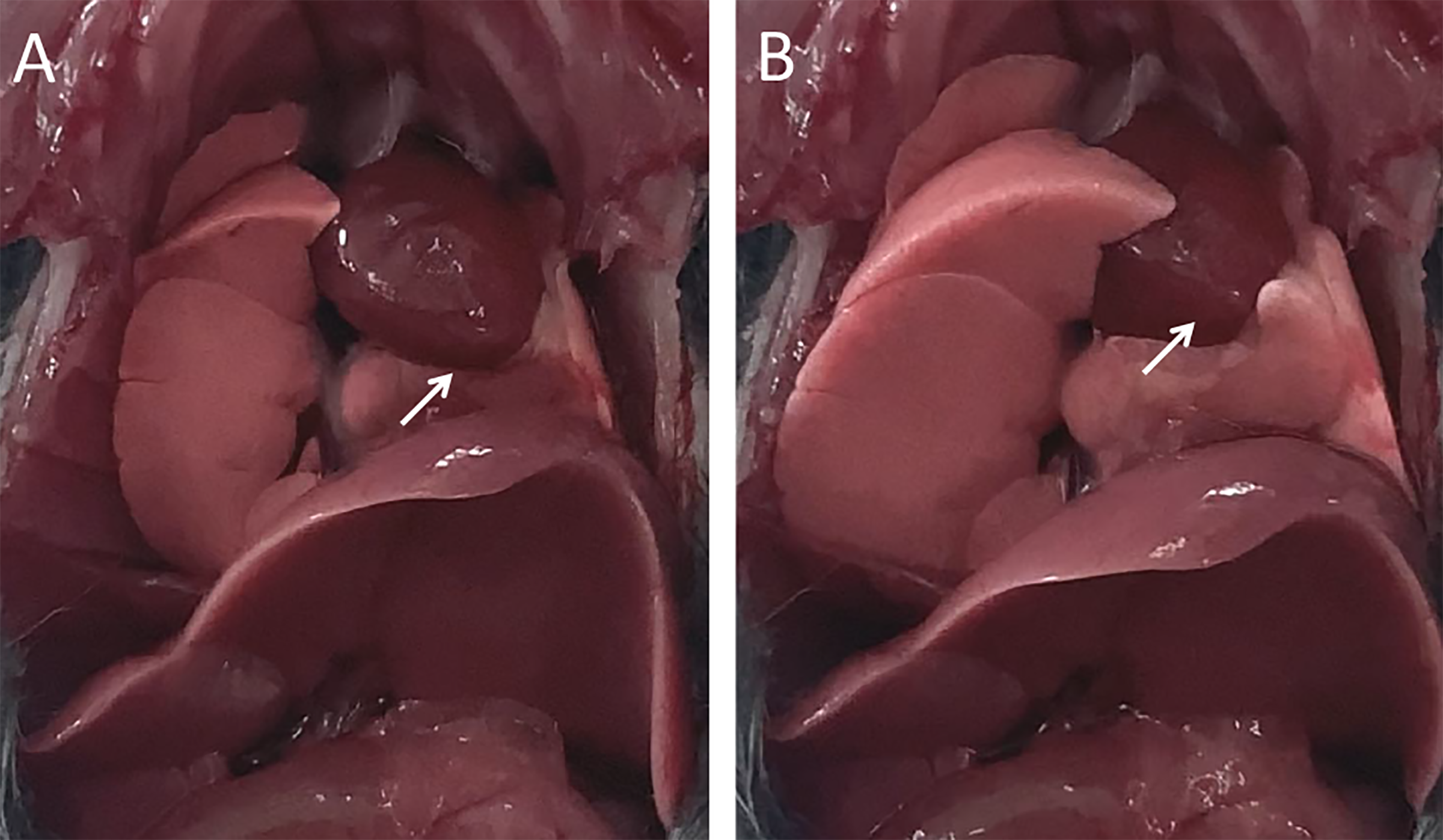

Supplement: Supplementary Figure 2 — The injection site during respiration. The heart was exposed during respiration, and part of the left ventricular tissue was uncovered by the lungs at both end-expiratory phase (A) and end-inspiratory phase (B). The injection site is indicated by the arrow. [file Image_2.TIF]

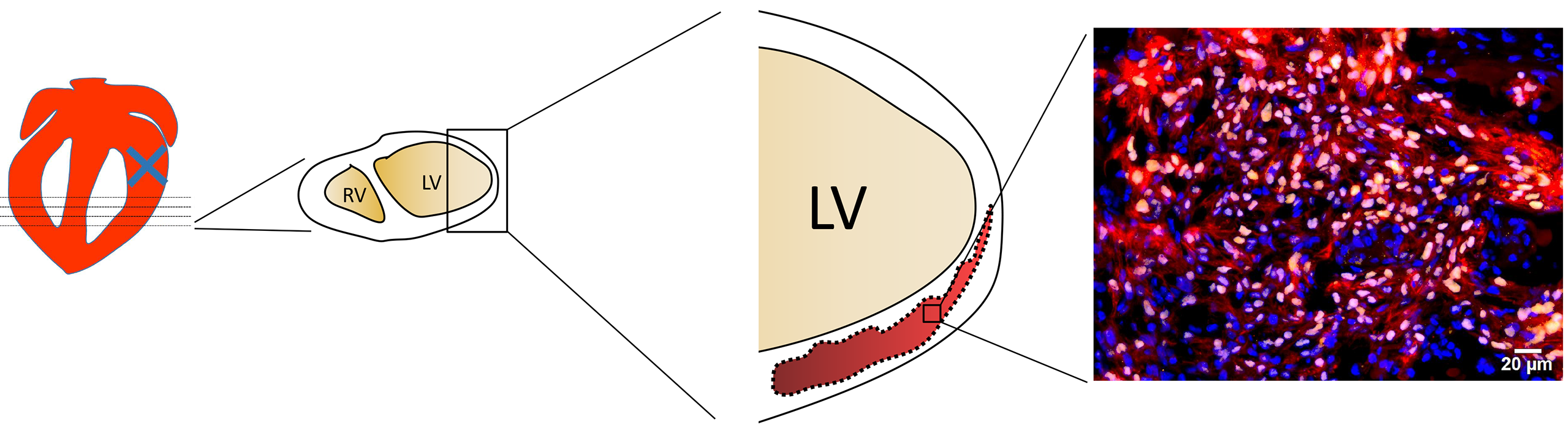

Supplement: Supplementary Figure 3 — Schematic diagram for the grafted cell number counting. [file Image_3.TIF]
